# A Qualitative Analysis of the Relationship Between Older Adults’ Daily Lives and Life Outlook

**DOI:** 10.1093/geroni/igaa057.2055

**Published:** 2020-12-16

**Authors:** Isabella Bouklas, Giancarlo Pasquini, Renee Gilbert, Cindy Bergeman, Stacey Scott

**Affiliations:** 1 Stony Brook University, Stony Brook, New York, United States; 2 University of Notre Dame, Notre Dame, Indiana, United States

## Abstract

Leading theories of adult development suggest age-related changes in one’s life perspective and changes in one’s priorities are reflected in daily behavior. The present study explored how older adults understand their current lives through a qualitative study of midwestern Americans. Twenty-four participants (Mage= 69.53 years; age range=63-78 years) from the Notre Dame Study of Health & Well-Being (Whitehead & Bergeman, 2014) completed semi-structured interviews in which they were asked about turning points across their lives. Inductive analysis using the constant comparative method (Maykut & Morehouse, 1994) resulted in 10 life-domains based on common descriptions across participants. These domains represented the ways in which participants understood their identities over the course of their lives, as well as their organization and use of time and space in daily life. Participants’ descriptions of both general life outlook and daily life informed one another, revealing the dialectical relationship between micro-level behaviors and macro-level attitudes.

